# Research Progress with Atractylone as an Antitumor Agent

**DOI:** 10.3390/molecules29225450

**Published:** 2024-11-19

**Authors:** Ying Yao, Guanghuan Shen, Jianghan Luo, Jinhong Wang, Zheng Xu, Hao Wang, Linlin Cui

**Affiliations:** 1 College of Pharmacy, Harbin University of Commerce, Harbin 150076, China; yy13087227552@163.com (Y.Y.); shengh@hrbcu.edu.cn (G.S.); 102690@hrbcu.edu.cn (J.L.); lilac241@163.com (J.W.); 18843672336@163.com (Z.X.); 2 Heilongjiang Provincial Key Laboratory of Geriatric Medicine, Harbin 150076, China; 3 Postdoctoral Programme of Meteria Medica Institute, Harbin University of Commerce, Harbin 150076, China

**Keywords:** atractylodes sesquiterpenes, anticancer, intestinal flora, mechanism, tumor treatment

## Abstract

Atractylone is a sesquiterpenoid compound extracted from *Rhizoma Atractylodis*. As one of the main active components in the volatile oil of the *Atractylodes* genus, it has exhibited certain therapeutic effects, including anti-inflammatory, antiviral, antioxidant, antiallergic, antiangiogenic, and neuroprotective activities, among others. With further research on the chemical constituents and pharmacology of sesquiterpenes, research on the antitumor activity of Atractylone has also been further expanded. Much of the current literature pays particular attention to the antitumor activity of Atractylone, which was found to inhibit the apoptosis of tumor cells and prevent growth, invasion, and migration through different apoptosis pathways and signaling pathways. Due to its promising potential for cancer prevention, it may play a role in reducing the incidence of malignant tumors. In this paper, the antitumor activity and mechanism of Atractylone are reviewed, providing a reference to inform future research on the tumor treatment, clinical application, and further development and utilization of this plant genus.

## 1. Introduction

At present, the treatment and rehabilitation of tumors is still a worldwide medical problem, with the difficulty and complexity of the treatment of advanced tumors placing doctors and patients in a difficult position for a long time [1,2]. The prevailing paradigm in modern medicine regarding tumor treatment emphasizes the attainment of a “tumor-free state”, primarily aimed at the complete eradication of tumor cells. While this approach has yielded significant therapeutic success, it has also resulted in irreversible damage to patients’ bodies [3,4]. Compared with traditional chemotherapy, the natural drug treatments have fewer side effects, less toxicity, and a milder curative effect, which can target multiple targets in tumor cells to affect multiple processes of tumorigenesis and development, and, thus, show great potential in tumor therapy.

*Rhizoma Atractylodis*, derived from the dried rhizome of *Atractylodes lancea (Thunb.) DC* and *Atractylodes chinensis (DC.) Koidz*. [5], has multiple pharmacological effects, such as antitumor, anti-inflammatory, anti-injury, antiallergy, and antiviral properties, as well as regulation of the gastrointestinal tract and protection of the cardiovascular and nervous systems [6,7,8,9,10]. As one of the traditional natural Chinese herbal medicines, it has a long history in clinical applications and is still in use today. While there is little variation between the main chemical components of the rhizome of *Atractylodes*, differences in their content lead to diverse pharmacological effects [11,12]. Atractylone, a sesquiterpene compound mainly extracted from *Rhizoma Atractylodis*, is one of the main active components in the volatile oil, accounting for up to 26.3% of the essential oil [13,14]. It exhibits a wide range of pharmacological effects, such as anti-inflammatory, antiallergic, antiviral, antioxidant, and neuroprotective activities, and has been reported to possess great potential in the field of tumor therapy. This paper reviews the progress of the antitumor activity and mechanisms of Atractylone in recent years, providing a reference and basis for its clinical application and the further development and utilization of this plant.

## 2. Atractylone and Its Antitumor Activity

**Figure 1:**
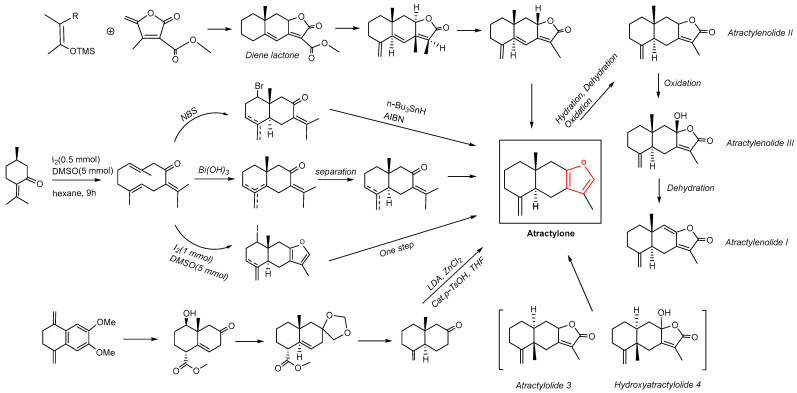
Chemical synthesis of Atractylone.

With the development of modern separation technology, many ketone compounds have been identified in *Atractylodes* [15,16]. Among them, Atractylone is the main compound used to distinguish genuine and nongenuine *Atractylodes* plants [17]. It is a natural furan compound basically separated from the rhizomes of *Rhizoma Atractylodis* and *Rhizoma Atractylodis Macrocephalae*, which is colorless to yellow liquid with a distinctive aromatic smell. Its molecular formula is C_15_H_20_O; its structure is shown in Figure 1. The furan ring structure, highlighted in red in the figure, is the basis of its antitumor effect, which can cause the generation of free radicals, DNA damage, and the inhibition of DNA generation in tumor cells [18]. Structural modification, by adding a furan ring to a natural compound, can enhance the antitumor activity and reduce the toxicity of the compound [19,20]. As a natural drug source, it has a potential inhibitory effect on diverse cancers, including leukemia [21], and its comprehensive toxicity risk profile presents no obvious adverse reactions. Its low toxicity, high content, and multiefficacy make it one of the most promising natural anticancer drugs, with a promising future in the field of cancer therapy.

**Figure 2:**
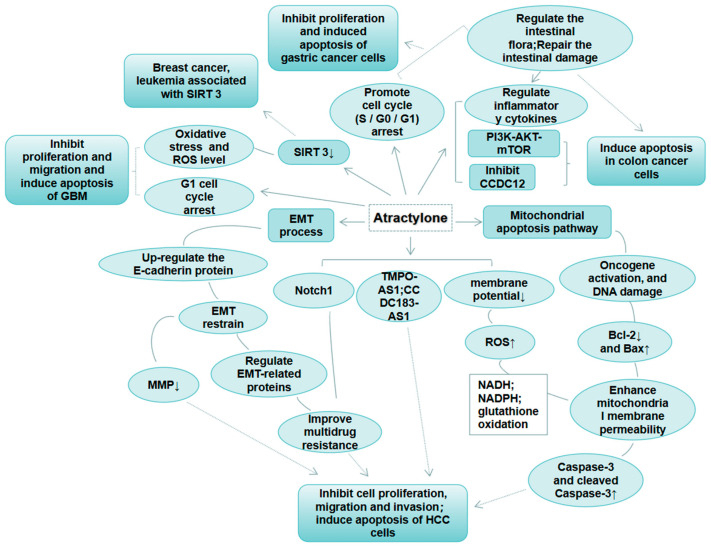
Mechanism of Atractylone on various tumor cells.

However, the presence of double bonds in the skeleton of Atractylone reduces its stability, making it unstable at room temperature and prone to self-oxidation under sunlight [22]. The decrease in effective content caused by this instability greatly weakens its biological efficacy. Thus, determining how to improve its stability and maintain its content and effective concentration is an urgent problem to solve. Studies have indicated that Atractylone can regulate apoptosis through different pathways and targets, as shown in Figure 2, and can also regulate the level of inflammatory response to inhibit the vitality of cancer cells and exert antitumor effects. A detailed discussion can be found in Table 1. The antitumor mechanisms are complicated and diverse, with a lot of targets that still need further exploration.

### 2.1. Anti-Liver-Cancer Activity

Liver cancer is one of the most common malignant tumors, and the morbidity and mortality rank high. The risk factors for liver cancer include chronic hepatitis, alcohol addiction, metabolic liver disease (mainly nonalcoholic fatty liver disease), and exposure to dietary toxins such as aflatoxin and aristolochic acid [23,24]. Early monitoring and detection can increase the potential for curative treatment [25].

**Figure 3:**
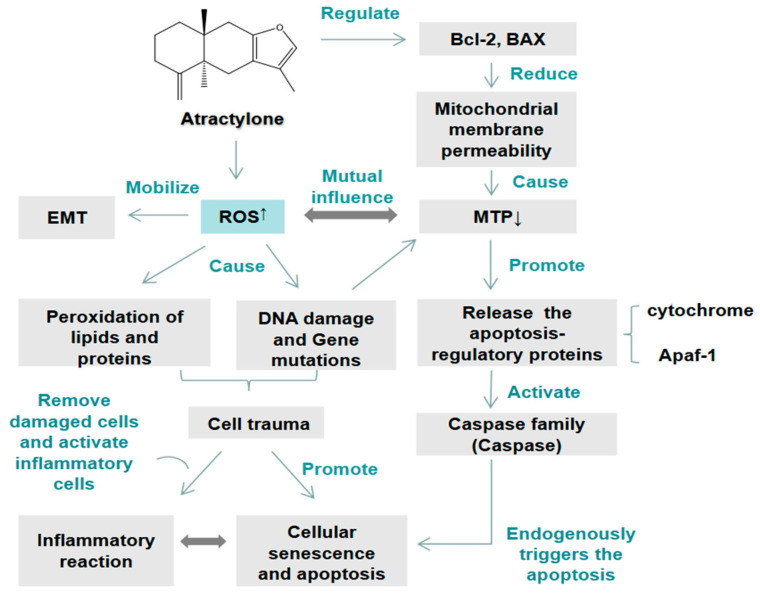
Mechanism of Atractylone on ROS channel in human liver cancer cells.

As a natural source of anticancer drugs, Atractylone was reported to inhibit the proliferation and growth of liver cancer cells effectively and played a crucial role in the early treatment of liver cancer. Cheng et al. [26] found that Atractylone can induce apoptosis of hepatocellular carcinoma cells (HCCs) through the mitochondrial apoptosis pathway. It can decrease the mitochondrial transmembrane potential (MTP), increase the level of reactive oxygen species (ROS), downregulate the expression of antiapoptosis factor Bcl-2, and upregulate the expression of apoptotic factors Bax and Cleaved caspase-3, ultimately resulting in endogenous cell apoptosis. MTP was the earliest event in the apoptotic cascade, causing a series of biochemical changes in the mitochondrial membrane [27] and thereby further increased the production of ROS. Simultaneously, excessive ROS can also affect MTP and damage cell proteins, lipids, and DNA, leading to cell damage, cell aging, and various forms of cell death, as shown in Figure 3. Cheng et al. continued their research through high-throughput sequencing. They found that Atractylone can regulate the expression of long-chain noncoding RNA TMPO-AS1 and long-chain noncoding RNA CCDC183-AS1. TMPO-AS1 was upregulated in HCC tissues and cells, and its depletion inhibited the proliferation and invasion of HCCs in vitro, as well as tumor growth and metastasis in vivo [28,29]. Therefore, TMPO-AS1 and CCDC183-AS1 were potential targets for the diagnosis and treatment of liver cancer [30]. Yang et al. also found that Atractylone can significantly inhibit the expression of Bcl-2, promote the expression of Bax and Cleaved caspase-3, and represent migration to induce apoptosis in HCCs [31], which provided a specific reference for further development and utilization of Atractylone. The inhibitory effect of Atractylone on HCCs may also be related to abnormal cell cycle regulation, and the intensity of action was obviously dose-dependent [32]. 

Atractylone was also responsible for inhibiting the migration and invasion of HCCs by upregulating epithelial markers (E-cadherin), preventing epithelial–mesenchymal transformation (EMT), and reducing the expression of matrix metalloproteinase MMP [27,30,31,33]. Multidrug resistance is known as the most common cause of tumor chemotherapy failure. Due to the high heterogeneity of tumor tissue, hepatocellular carcinoma patients often develop resistance to chemotherapy drugs and targeted drugs. EMT transcription factors, cytokines, associated signaling pathways, and some noncoding RNA during EMT transformation mediated the drug resistance in HCCs. It was suggested that Atractylone could regulate the EMT process and additionally regulate EMT-related proteins, reduce intercellular adhesion, change cell morphology, and enhance cell migration and invasion ability, significantly improving drug resistance of HCCs [34,35,36]. Tang et al. explored the action of Atractylone on chemotherapy resistance of HCCs based on the Notch 1 pathway. They discovered that Atractylone downregulated the expression of Notch1, Hes1, Jagged1, and Bcl-2, upregulated the expression of Bax, promoted the apoptosis of Bel/Fu cells, inhibited the survival and invasion, and enhanced the chemosensitivity of HCCs. The mechanism was correlated with the repression of the activation of the Notch 1 pathway. The Notch 1 pathway was closely linked with various signal pathways involved in tumorigenesis. It was widely involved in the occurrence and development of many kinds of malignant tumors [37]. Therefore, Atractylone was expected to be an inhibitor of the Notch1 signal pathway. To sum up, the antihepatoma activity of Atractylone not only regulated the expression of related apoptotic proteins through related apoptotic pathways, but also regulated the EMT process of cancer cells and improved the multidrug resistance of HCCs by inhibiting the Notch 1 pathway to inhibit the cell growth, migration and invasion, and achieve an antihepatoma effect.

### 2.2. Anti-Colon-Cancer Activity

Colon cancer is one of the common malignant tumors that seriously endanger public health. In recent years, the incidence of colon cancer in people under the age of 50 was increasing yearly. It was generally thought to be caused by random genetic mutations. Moreover, smoking and unhealthy diets also increase the risk of colon cancer in humans [38,39]. Therefore, the prevention, early detection, and improvement of the curative effect of colon cancer are essential. Determining how to solve the problem of the increasing number of young colon cancer patients and find better treatment and drugs is an urgent task.

Buris et al. [40] confirmed that the activation of the PI3K/AKT/mTOR signaling pathway mediated by molecular aberrations contributed to tumor development and resistance to anticancer therapy, suggesting that the PI3K/AKT/mTOR signaling pathway was an essential target for the treatment of colon cancer. Geng et al. [41] found that Atractylone inhibited the proliferation of the colorectal cancer HT-29 cell line in a dose-dependent manner. The expression of curly helix domain 12(CCDC12) gene, PI3K, pAKT and mTOR genes, and proteins in HT-29 cells was decreased after the treatment of Atractylone, indicating that Atractylone can promote the apoptosis of colorectal cancer cells by inhibiting the CCDC12 and regulating the PI3K/AKT/mTOR signal pathway of HT-29. Mao et al. [42] then continued exploring the effects of Atractylone on the proliferation and apoptosis of colorectal cancer cells through the PI3K-AKT-mTOR signal pathway and found that under the treatment of Atractylone of 30 mg/mL, the expression of INF-γ, TNF-α, and MMP-9 were observably decreased, the expression of Bcl-2, PI3K, AKT, and mTOR was significantly reduced, and the Caspase-3 was increased, which further indicated that proper concentration of Atractylone can inhibit the proliferation and promote apoptosis of colon cancer cells by inhibiting the PI3K/AKT/mTOR signal pathway, which was conducive to enhancing the repair of intestinal epithelial barrier function and provided a specific clinical reference for the treatment of colon cancer. As described above, the PI3K/AKT/mTOR pathway may be a key target for treating intestinal cancer, and Atractylone could be an effective candidate drug for HT-29 cells.

The incidence of colon cancer was the result of the joint action of multiple factors, such as host and environment [43]. It was found that diet, microorganism group, obesity, and family history were some etiological factors leading to the occurrence of colon cancer [44]. Among them, intestinal microorganisms had a particular indicator effect on the therapeutic effect of diseases. The structure and function of intestinal microorganisms were intimately related to the occurrence, diagnosis, and treatment of colorectal tumors, which was an essential factor in the development of ulcerative colitis (UC). Therefore, detecting intestinal microorganisms was the way to improve the early diagnosis rate of intestinal tumors, as the intervention of microflora by diet control may be a crucial part of colon cancer prevention and treatment strategy [45,46]. In addition, inflammatory factors play a central role in the occurrence and development of cancer [47,48,49]. Li et al. [50] found that Atractylone, as the main active component of the essential oil of *Atractylodes lancea*, can relieve the symptoms of UC in vivo. It can reduce tumor necrosis factor (TNF)-α and reactive oxygen species (ROS) and increase the expression of adhesion proteins such as claudin, ZO-1, and occludin. At the same time, the analysis of metabolic pathways showed that the effect of Atractylone mainly focuses on the amino acid metabolism pathway, which was conducive to repairing intestinal damage and improving intestinal immunity. The imbalance of intestinal flora and development of malignant tumors may be linked to intestinal dysbacteriosis, the production of pathogenic microorganisms, microbial toxins and toxic factors, and the abnormal metabolism regulated by flora [51], so that regulating the balance of intestinal flora was an essential means to prevent the development of tumor. Atractylone was accurately found to restore intestinal function by regulating the inflammatory response caused by microbiosis. Treatment and prevention can prevent UC turning into colorectal cancer.

### 2.3. Anti-Glioblastoma Activity

Glioblastoma (GBM) is the most common pathological type of primary malignant nervous system tumor [52]. At present, there are a variety of treatments for glioblastoma, including surgery, radiotherapy, systemic therapy (chemotherapy and targeted therapy), and supportive therapy. Recently, tumor treating electric fields (TTFields) therapy, a new therapy for glioblastoma (GBM), has gradually sprung up [53]. Despite active adjuvant therapy after operation, the prognosis was still poor and the average survival rate of patients was still meager.

Atractylone has been proven to have a definite effect on GBM cells, and researchers have made a breakthrough in exploring its mechanism. Sun et al. [54] explored the effects of Atractylone on the proliferation, migration, and apoptosis of GBM cell lines C6 and DBTRG. They discovered that it reduced the viability of GBM cells in a dose- and time-dependent manner, inhibited the proliferation and migration of GBM cells, and induced apoptosis by causing G1 cell cycle arrest in GBM cells. The associated mechanism was sirtuin 3 (SIRT 3), a tumor suppressor. Previous studies proved that SIRT 3 has been a significant focus of cancer research, which plays a central role in mitochondrial biology and can promote cell survival by regulating oxidative stress pathways while maintaining ROS levels and proliferative and invasive phenotypes. Consequently, this regulation helps prevent apoptosis and facilitates carcinogenesis [55]. Atractylone can effectively inhibit the proliferation of gastric cancer cells and induce apoptosis of gastric cancer cells. Early studies also found that it can promote the expression of SIRT3 [56] and inhibit a variety of cancer cells. In the experiment that treated C6 cells with Atractylone, the protein level of SIRT 3 and the signal intensity of immunostaining SIRT3 increased significantly. The mRNA level of SIRT 3 in cells also increased, which showed that Atractylone can activate SIRT 3 signaling in vitro and in vitro to inhibit tumor occurrence of GBM cells and exert its activity against GBM cells. Atractylone also acted on the Notch1 pathway and was central in GBM tumor growth, angiogenesis, and radioresistance [57,58]. This finding may be favorable for promoting the design of more effective therapies targeting Notch 1 for GBMs.

### 2.4. Anti-Gastric-Cancer Activity

Wang et al. [59] investigated the proliferation inhibition effect of the extract of *Atractylodes lancea* on BGC-823 and SGC-7901. They found that the cell cycle of BGC-823 was arrested in the S phase and that of SGC-7901 in the G_0_/G_1_ phase, which could effectively inhibit the proliferation of gastric cancer cells and induce apoptosis of gastric cancer cells. Consequently, the detailed mechanism of action of Atractylone within the gastric cancer cell extract has yet to be thoroughly investigated. The results of in vitro cytotoxicity experiments performed by Gu S. et al. [60] on volatile oil-derived substances such as Atractylone indicated that it could inhibit cell proliferation such as MCG803, HepG2, and HCT-116, showing significant anti-gastric-cancer cell activity. However, the specific mechanism has not been defined.

**Figure 4:**
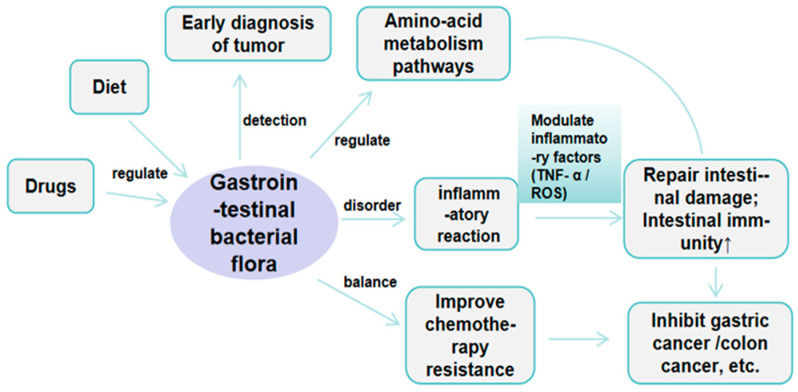
Association between gastrointestinal tract flora and tumor development.

Intestinal microorganisms were found to be an essential factor affecting the development of colon cancer in the study of Atractylone against colon cancer. Similarly, gastric microflora was also an important factor affecting the treatment of gastric cancer. Chemotherapy was one of the critical means in the treatment of gastric cancer, especially in the treatment of advanced gastric cancer. In addition to the direct effect of chemotherapy on cancer cells, it may also affect the microecosystem in the human body, especially the microflora of the gastric environment. The type and number of microorganisms in the gastric environment may regulate the level of inflammation in the body. Moderate inflammation was beneficial in guiding the immune system to attack cancer cells and regulating the balance of microbial flora, resulting in improved tolerance to chemotherapy and efficacy [61,62], as shown in Figure 4. Li et al. [63] found that Atractylone can reduce the production of inflammatory factors and oxidative stress, regulate inflammation-related intestinal microbiota disorders in the treatment of gastrointestinal diseases, and play a significant role in the treatment of gastric ulcer, which also suggests that Atractylone has a potential role in the treatment of gastric cancer.

### 2.5. Activity Against Other Cancer Cells

Wang et al. [64] proposed the dose-dependent growth inhibition of Atractylone on human leukemia cell line HL-60 and mouse leukemic cell line P-388. Through the study of cytotoxicity in vitro, Atractylone showed a strong antiproliferation effect on HL-60 and P-388. The specific experiment showed that treatment under the action of Atractylone of 15 μg/mL for 6 h induced apoptosis in HL-60 cells, and, compared to other compounds of the same type, it induced shorter apoptosis and was less cytotoxic to normal human peripheral blood mononuclear cells. Nevertheless, so far, the mechanism of antileukemic cell proliferation of Atractylone is still unclear, and further exploration is needed. Yu et al. [65] isolated Atractylone from *Atractylode* extract and found that it can inhibit Tissue Plasminogen Activator (TPA)-induced inflammation in mice and significantly inhibit tumor growth in mice. Compared with standard drugs, its antitumor effect was similar to that of Cephalosporin and Azaphenanthroline, but more effective than Quercetin (a known tumor progression inhibitor). It was consequently found to inhibit the tumor-promoting effect of two-stage carcinogenesis in the mouse skin. SIRT3 has been proved to be a tumor suppressor gene of breast cancer cells, such as breast cancer [66], leukemia [67], and liver cancer [68]. Therefore, except for liver cancer and leukemia, Atractylone may also have a specific action on breast cancer cells, and the mechanism and therapeutic effect still need to be studied further as well.

## 3. The Chemical Synthesis of Atractylone

In 1980, Arthur et al. described the total synthesis of furanosesquiterpene df-Atractylone in detail, and diene lactone served as an intermediate in this process. The annelation approach to eudesmane sesquiterpenes has been highly effective [69]. Jonida et al. found a sustainable approach to chemical synthesis: I_2_/DMSO-mediated oxidative C–C and C– heteroatom bond formation. The system I_2_/dimethyl sulfoxide mediated the one-step transformation of α-isopropylidene ketones into furan rings following a biomimetic approach [70]. This methodology has been used to synthesize terpene furans, including Atractylone. I_2_/DMSO was a thriving catalytic system that prepares C–C and C–X (X = O/S/N/Se/Cl/Br) bonds, forming various bioactive molecules [71]. Studies have reported that the reductive alkylation of α-tetralone derivatives leads to suitably functionalized decalins, which can synthesize germacrene sesquiterpenes. This method can also be applied to the total synthesis of Atractylone [72]. Atractylone has also been shown to be a possible intermediate during the biosynthesis of AtractylenolideI, II, and III and can be transformed by oxidation from Atractylone [73]. Moreover, Atractylolide 3 and Hydroxyatractylolide 4 could emanate in vivo from Atractylone [74], as shown in Figure 1.

As mentioned above, the presence of double bonds in the skeleton of Atractylone reduces its stability, rendering it unstable at room temperature and making it susceptible to auto-oxidation under sunlight. This instability’s decrease in effective content significantly diminishes its biological efficacy. There is limited research on its stability, and structural modifications aimed at enhancing both stability and bioavailability represent a promising area for future investigation. Under conditions where the antitumor properties of the structure remain unaffected, efforts can be directed toward modifying the double bonds to improve stability and reduce auto-oxidation potential, thereby achieving optimization objectives.

## 4. Discussion

As one of the main active components of sesquiterpenes of *Atractylodes* genus plants, Atractylone has effects such as antitumor, anti-inflammation, antivirus, antioxidation, antiallergy, stomach protection, anti-blood-tube formation, neuroprotective activity, etc. Current studies have found that the potential antitumor targets of Atractylone are AKT, MMP-9, Bcl-xl, dipeptidyl peptidase IV, retinoic acid β receptor, cellular retinoic acid binding protein 2 [75,76], TMPO-AS1, CCDC183-AS1, and so on. It can activate the caspase-mediated apoptosis pathway, regulate the production of ROS, the balance of microbial flora and inflammatory factors in the gastrointestinal tract, and the EMT process to improve cell drug resistance while activating the related Notch1 pathway and downregulating PI3K/AKT/mTOR signaling pathway to mediate apoptosis and promote cell cycle arrest in different periods to induce cancer cell apoptosis, as shown in Table 1. It shows the inhibitory effect and mechanism of Atractylone on the growth and proliferation targets and pathways of cancers. It reveals the potential of Atractylone in the treatment of cancer. In addition to inducing apoptosis and inhibiting the growth, migration, and invasion of cancer cells through apoptosis pathways and some signal pathways, it is worth paying attention to the effect of microbial environment on some cancers. There is also a particular relationship between the microbial environment and the level of inflammatory cytokines, which is essential in tumor formation and development. Thus, future studies could focus on in vivo microbial modulation of associated inflammatory factors to inhibit cancer cell development. Identifying beneficial symbiotic gut microbiota will be a therapeutic target for inflammation-mediated gastrointestinal cancer. Therefore, the intervention of microflora through diet and drugs may become one of the strategies for cancer prevention and treatment.

This review can provide a theoretical basis for the antitumor application of Atractylone. However, due to the complex and diverse mechanisms of different carcinogenesis involving multiple targets and pathways, the antitumor activity of Atractylone and its possible mechanisms still warrant further experimental verification. Furthermore, the clinical study on the drug toxicity of sesquiterpenes of Atractylone is still very shallow. At the same time, attention should be paid to the chronic and long-term toxicity risk caused by the clinical use of Atractylone in the treatment of many kinds of cancer and the toxic reactions caused by the interaction of metabolic coadministration drugs. In order to maximize the clinical application potential of Atractylone and fully explore its potential molecular mechanism in the treatment of various cancers, large-scale and multicenter cooperative clinical trials are urgently needed in the future.

**Table 1:** The MOAs for Atractylone’s tumor growth inhibition for targets and pathways.

Pathways and Targets	Mechanism of Action (MOA)
Caspase-mediated apoptosis pathway	Abnormal reduction in apoptosis is a significant factor contributing to tumorigenesis. The imbalance between proapoptotic and antiapoptotic proteins plays a crucial role. Atractylone has been found to downregulate Bcl-2 and upregulate Bax and Cleaved caspase-3, ultimately leading to intrinsic cellular apoptosis. Caspases are essential in initiating and executing apoptosis. Their reduced activity can result in decreased apoptosis and abnormal signaling pathways. The signaling pathways mediating apoptosis include extrinsic pathways mediated by death receptors and intrinsic pathways mediated by mitochondria [77]. Atractylone primarily targets the intrinsic pathway, which relies on the release of specific active substances from mitochondria. Thus, this apoptotic pathway is referred to as mitochondrial apoptosis. The intrinsic pathway is triggered upon internal stimulation, resulting in the opening of the mitochondrial permeability transition pore and loss of the mitochondrial transmembrane potential. As an outcome, several proapoptotic proteins, such as cytochrome c, are released into the cytoplasm, eventually activating caspase. Though different pathways may initiate apoptosis, cell death is induced through the execution pathway, which involves caspase-3 activation [78,79].
ROS	ROS is a byproduct of normal cellular metabolism, which contains derivatives comprising highly unstable oxygen free radicals, such as superoxide anion (O_2_^•−^) and hydroxyl (OH•). Mitochondria and NADPH oxidases are two significant contributors to endogenous ROS in cancer [80]. Atractylone affects hepatocellular carcinoma cells by inducing apoptosis via the mitochondrial apoptotic pathway, which characterizes a reduction in MTP and an elevation in ROS levels. The implications of increased ROS levels can be multifaceted, as they may both promote and inhibit malignant behavior. Elevated ROS levels are considered oncogenic due to their capacity to oxidize nucleic acids and cause damage to DNA, proteins, and lipids. Furthermore, Atractylone functions as a potential antioxidant by facilitating the degradation of superoxide anion (O_2_^•−^) and hydrogen peroxide (H_2_O_2_), thereby preventing oxidative damage to DNA. Additionally, Atractylone has been shown to diminish the production of inflammatory factors and oxidative stress in the gastrointestinal tract.
MMPs	Matrix metalloproteinase-9 (MMP-9), whose expression is frequently dysregulated in cancer, promotes tumor growth, invasion, and metastasis by multiple mechanisms. MMPs have long been associated with solid tumor invasion, metastasis, and angiogenesis [81]. MMP-9 regulates tumor growth, invasion, and metastasis, including proteolytic remodeling of extracellular matrix (ECM), alteration of cell–cell and cell–ECM interactions, migration, and angiogenesis [82]. After treatment, Atractylone significantly reduces the expression of INF-γ, TNF-α, and MMP-9 in colorectal cancer cells. MMPs are zinc-dependent endopeptidases that play a crucial role in the degradation and remodeling of ECM components and basement membranes. Overexpression of MMP genes can lead to ECM remodeling, thereby enhancing cancer cells’ invasive and migratory capabilities. Conversely, inhibiting the expression of MMP-2 and MMP-9 can decrease cancer cell adhesion and metastasis [83,84]. Therefore, targeting the suppression of MMP expression presents an effective strategy for controlling cancer cell metastasis.
AKT	AKT is a promising target and an effector of the PI3K/AKT/mTOR pathway that activates tumors. The AKT kinase family comprises the AKT1, AKT2, and AKT3 isoforms. AKT activity is controlled in an AKT-dependent manner via phosphorylation and dephosphorylation [85]. The PI3K/AKT/mTOR axis is significantly altered in cancer, and targeting this axis with multiple inhibitors can modulate a variety of cellular processes such as cell proliferation, autophagy, apoptosis, angiogenesis, EMT, and chemoresistance [86]. The PI3K/AKT/mTOR pathway is a key target for treating colorectal cancer, and after treatment with Atractylone, the expression of PI3K, AKT, and mTOR in colorectal cancer cells is significantly reduced. Aberrant activation of this pathway induces cell survival and metastasis. It can also lead cancer cells to escape from apoptosis through dysregulation of anti- and proapoptotic genes. It also possesses a central role in EMT promotion and chemoresistance [87]. Targeted therapy against the PI3K pathway may be an essential strategy in treating colorectal cancer.
Arresting the cell cycle	The process of cell division plays a vital role in cancer progression. Cell proliferation and error-free chromosome segregation during mitosis are central events in the life cycle. Mistakes during cell division generate changes in chromosome content and alter chromosome number balances [88]. Cancer cells are characterized by their ability to proliferate and divide indefinitely, evading regulation by the cell cycle. This uncontrolled proliferation is a hallmark of dysregulated cell cycles. Cancer cells have evolved mechanisms that allow them to alter their structure and bypass signaling pathways and checkpoint controls to escape surveillance by the immune system and circumvent regulatory checkpoints in the cell cycle. These adaptations ensure genomic stability in cancer cells while promoting unrestrained growth. Consequently, targeting the cell cycle has been regarded as a promising strategy for cancer therapy [89]. One approach within cell-cycle-targeted therapy involves controlling the expression of cyclins and cyclin-dependent kinases (CDKs), or other molecules associated with abnormal checkpoint regulation, to manage cancer cell growth [90]. Atractylone has been shown to inhibit the proliferation and migration of glioblastoma (GBM) cells while inducing apoptosis through G1 phase arrest in these cell. Further research is needed to explore its significant potential in targeted therapies against cancer related to the cell cycle.
Microbial flora	Gastrointestinal microorganisms have been identified as significant factors influencing cancer development in studies examining the effects of Atractylone on colon and gastric cancer. Inflammatory factors play a central role in the occurrence and progression of cancer. Atractylone has been shown to reduce the production of inflammatory factors and oxidative stress within the gastrointestinal tract. This is achieved by lowering tumor necrosis factor (TNF)-α and ROS levels while simultaneously increasing the expression of adhesion proteins such as claudin, ZO-1, and occludin. These actions help regulate inflammation-related dysbiosis in gut microbiota when treating gastrointestinal diseases. The effects of Atractylone primarily focus on amino acid metabolic pathways, which are beneficial for repairing gastrointestinal damage and enhancing intestinal immunity.
Epithelial–mesenchymal transition (EMT)	During the EMT transformation process, EMT transcription factors, cytokines, related signaling pathways, and some noncoding RNAs mediate the resistance of HCCs. Atractylone can regulate the EMT process and EMT-related proteins, reduce cell-to-cell adhesion, change cell morphology, enhance cell migration and invasion ability, and significantly improve the resistance of HCCs. EMT is a switch that allows cells to transition between epithelial and mesenchymal states and is a key to the metastatic process [91].
TMPO-AS1 and CCDC183-AS1	Atractylone has been shown to regulate the expression of TMPO-AS1 and CCDC183-AS1, inhibit the proliferation, invasion, and migration of HepG2 hepatocellular carcinoma cells, and improve cell apoptosis, making it a potential target for the treatment of liver cancer with Atractylone. LncRNA TMPO-AS1 can regulate the progression of the cancer cell cycle and adhesion, and further affect the prognosis of patients. CCDC183-AS1 can improve the proliferation, invasion, and migration of HepG2 cells treated with Atractylone and increase cell apoptosis [92,93,94].
SIRT3	SIRT3 has always been a focus of cancer research, playing a central role in mitochondrial biology. It can promote cell survival by regulating the oxidative stress pathway while maintaining ROS levels, proliferation, and invasive phenotypes to prevent cell apoptosis and promote carcinogenesis. The mechanism of action of Atractylone on SIRT3 is still not well understood and needs further research.
Notch 1	The Notch 1 pathway is closely related to various signaling pathways in tumor development. Atractylone has not yet deepened research on Notch 1. It acts on the Notch1 pathway, playing a core role in the growth of GBM tumors, angiogenesis, and radiation resistance. Notch ss actively involved in a process known as epithelial-to-mesenchymal transition (EMT), in which epithelial cells obtain a mesenchymal phenotype, ultimately leading to migration. This is achieved by the interaction of Notch with transcription factors Slug, Snail, and TGF-b, which are critical for EMT [95]. Notch signaling highly regulates the formation and maintenance of cancer stem cells (CSCs), which lead to metastasis and tumorigenesis.
Other targets	Dipeptidyl peptidase IV, retinoid acid β receptor, and cellular retinoic acid-binding protein 2 are also predictive targets for the anticancer effects of Atractylone. Specific studies have not yet been conducted, and this still needs further exploration.

## 5. Materials and Methods

A thorough literature search was carried out using different search engines, including Pubmed, CNKI, Google Scholar, SciFinder, Crossref, ScienDirect, and Baidu Scholar to select the publications describing the antitumor activity of Atractylone and its mechanism of action. We marked its provenance after the references, indicating where to find it. The main keywords we searched for were Atractylone, Atractylon, Atractylodes sesquiterpenes, anticancer, tumor treatment, etc. Most of the articles are less than 10 years old.

## Data Availability

The data used to support the findings of this study are included within the article.
